# Evolution of altitudinal migration in passerines is linked to diet

**DOI:** 10.1002/ece3.6126

**Published:** 2020-03-05

**Authors:** Claudie Pageau, Mariana M. Vale, Marcio Argollo de Menezes, Luciana Barçante, Mateen Shaikh, Maria Alice S. Alves, Matthew W. Reudink

**Affiliations:** ^1^ Department of Biological Sciences Thompson Rivers University Kamloops BC Canada; ^2^ Ecology Department Federal University of Rio de Janeiro Rio de Janeiro Brazil; ^3^ National Institutes for Science and Technology in Ecology, Evolution and Biodiversity Conservation Goiás Brazil; ^4^ Physics Institute Fluminense Federal University Niteroi Brazil; ^5^ National Institute of Science and Technology on Complex Systems Rio de Janeiro Brazil; ^6^ Programa de Pós‐graduação em Ecologia e Evolução Universidade do Estado do Rio de Janeiro Rio de Janeiro Brazil; ^7^ Department of Mathematics & Statistics Thompson Rivers University Kamloops BC Canada; ^8^ Departamento de Ecologia Universidade do Estado do Rio de Janeiro Rio de Janeiro Brazil

**Keywords:** bird movement, evolution, foraging guild, Passeriformes, phylogenetic comparative analysis

## Abstract

Bird migration is typically associated with a latitudinal movement from north to south and vice versa. However, many bird species migrate seasonally with an upslope or downslope movement in a process termed altitudinal migration. Globally, 830 of the 6,579 Passeriformes species are considered altitudinal migrants and this pattern has emerged multiple times across 77 families of this order. Recent work has indicated an association between altitudinal migration and diet, but none have looked at diet as a potential evolutionary driver. Here, we investigated potential evolutionary drivers of altitudinal migration in passerines around the world by using phylogenetic comparative methods. We tested for evolutionary associations between altitudinal migration and foraging guild and primary habitat preference in passerines species worldwide. Our results indicate that foraging guild is evolutionarily associated with altitudinal migration, but this relationship varies across zoogeographical regions. In the Nearctic, herbivorous and omnivorous species are associated with altitudinal migration, while only omnivorous species are associated with altitudinal migration in the Palearctic. Habitat was not strongly linked to the evolution of altitudinal migration. While our results point to diet as a potentially important driver of altitudinal migration, the evolution of this behavior is complex and certainly driven by multiple factors. Altitudinal migration varies in its use (for breeding or molting), within a species, population, and even at the individual level. As such, the evolution of altitudinal migration is likely driven by an ensemble of factors, but this study provides a beginning framework for understanding the evolution of this complex behavior.

## Graphical abstract

Globally, 830 of the 6,579 Passeriformes species are considered altitudinal migrants and this pattern has emerged multiple times across 77 families of this order. We investigated potential evolutionary drivers of altitudinal migration in passerines around the world by using phylogenetic comparative methods. Our results indicate that foraging guild is evolutionary associated with altitudinal migration, but this relationship varies across zoogeographical regions.

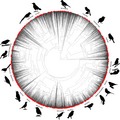

## 1 INTRODUCTION

**Figure 1:**
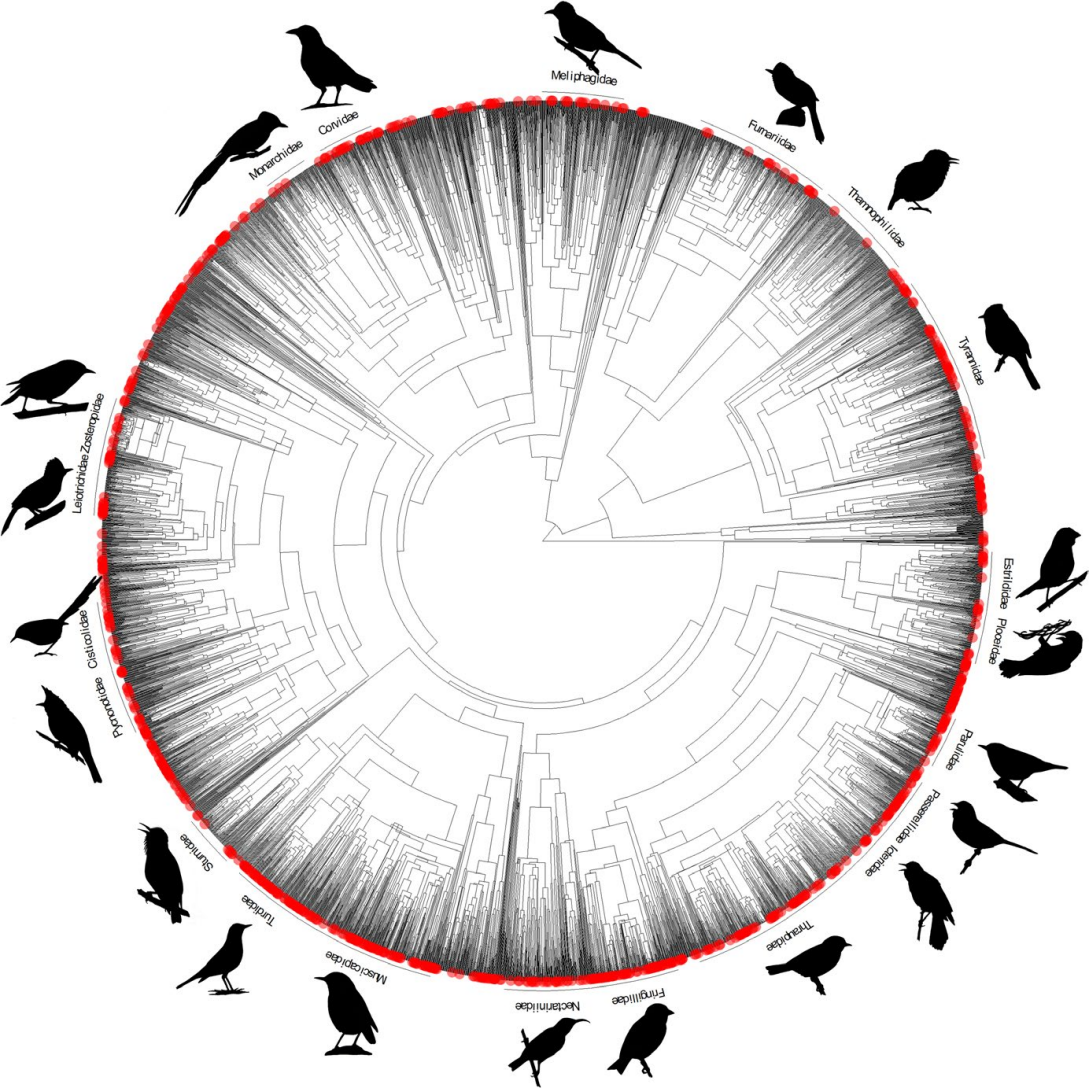
Phylogeny of all Passeriformes and occurrences of altitudinal migration represented by red circles. Speciose families' (>100 species) names and silhouettes are shown along the outside of the phylogeny

Altitudinal migration is generally described as a seasonal movement from lower elevations to higher elevations for the breeding season and a downslope movement for the nonbreeding season (Barçante, Vale, & Alves, 2017; Hayes, 1995; Mackas et al., 2010). Some species also engage in altitudinal movements to reach molting grounds (Rohwer, Rohwer, & Barry, 2008; Wiegardt, Wolfe, Ralph, Stephens, & Alexander, 2017). Altitudinal migration has been observed in a broad diversity of bird species; in total, 1,238 species across 130 families of birds have been described as altitudinal migrants (Barçante et al., 2017), suggesting repeated independent evolution of this behavior (Figure 1). There are three main advantages ascribed to altitudinal migration: reduction in the risk of predation (Boyle, 2008a), avoidance of harsh climatic conditions (Boyle, 2008b; Boyle, Norris, & Guglielmo, 2010; Hahn, Sockman, Nreuner, & Morton, 2004), and tracking of food resources (Chaves‐Campos, 2004; Kimura, Yumoto, & Kikuzawa, 2001; Levey, 1988; Loiselle & Blake, 1991; Solorzano, Castillo, Valverde, & Avila, 2000).

Most studies on altitudinal migration have focused on the food abundance hypothesis rather than predation and climatic conditions, which are extremely challenging to study across a wide range of species and habitats. Some studies on altitudinal migration have provided evidence that frugivorous bird abundance is linked to fruit and flower abundance (Chaves‐Campos, 2004; Kimura et al., 2001; Levey, 1988; Loiselle & Blake, 1991) while others have shown no evidence of this phenomenon (Boyle, 2010; Hart et al., 2011; Papeş, Peterson, & Powell, 2012; Rosselli, 1994). Boyle (2017), Chaves‐Campos (2004), Kimura et al. (2001) and Pratt, Smith, and Beck (2017) suggested that food abundance drives uphill migration only, but this might depend of the species since Loiselle and Blake (1991) described downhill movement for some frugivorous species in Costa Rica when food was decreasing.

If altitudinal migration evolved as a strategy to track food resources, we would predict a link between diet (foraging guild) and altitudinal migration; however, the evidence for this relationship remains unclear. Frugivory has been suggested as a driver of altitudinal migration, in part because frugivorous altitudinal migrants have been observed more frequently at higher elevations in Costa Rica (Blake & Loiselle, 2000; Boyle, Conway, & Bronstein, 2011) and Nepal (Katuwal et al., 2016). However, Barçante et al. (2017) examined the foraging guild of all altitudinal migrant birds and showed that invertivorous altitudinal migrants are most abundant worldwide, except in the Neotropics where frugivores and nectivores are more abundant. Despite the fact that insect abundance in temperate regions is often posited as a major driver of the evolution of long‐distance migration, little research has been dedicated to the role of insect abundance in the study of altitudinal migration even though insect intake might be crucial during the breeding season (Chaves‐Campos, 2004; Levey, 1988) and invertivore bird species have been shown to vary in elevation seasonally in mountainous environments, such as Nepal (Katuwal et al., 2016).

Altitudinal migration has been observed in every zoogeographical region in the world (Barçante et al., 2017) although some hotspots seem to host a higher proportion of altitudinal migrants, such as the Himalayas and western North America (Boyle, 2017). It is important to note, however, that some of this variation in the proportion of altitudinal migrants could result from a difference in sampling efforts across the world (Barçante et al., 2017). Alternatively, environmental conditions in those regions, such as habitat availability and seasonality, may also favor the evolution of altitudinal migration.

Our goal was to examine potential drivers of the evolution of altitudinal migration in passerines. The order Passeriformes represents approximately half of the avifauna and 13% of them are described as altitudinal migrants, making them a good choice for this study. Of the 6,579 passerines species and subspecies recorded in this study, 830 species are considered altitudinal migrants and are distributed across 77 of the 137 families of Passeriformes (Figure 1). Using a speciose and globally distributed group of birds, we conducted large‐scale phylogenetic comparative analyses to examine evolutionary associations between altitudinal migration and diet (foraging guild) and habitat. In addition, we asked whether these associations differ depending on the zoogeographic region. We expected that frugivorous and nectivorous species were driven toward altitudinal migration in the Neotropics because they were tracking fruit and flower abundance which varies seasonally (Barçante et al., 2017; Chaves‐Campo, 2004; Kimura et al., 2001; Levey, 1988; Loiselle & Blake, 1991). For every other region, invertivorous species would be driven toward altitudinal migration (Barçante et al., 2017). We also expected altitudinal migration to be evolutionary associated with forest habitats in the Neotropics because altitudinal migrants in Costa Rica (Stiles, 1988; Stiles & Clarke, 1989) and southeastern Brazil (Stotz, unpublished—see Stotz, Fitzpatrick, Parker, & Moskovits, 1996), for instance, include a high number of restricted‐range and forest‐dependent species.

## 2 METHODS

### 2.1 Ethics statement

No permits were required for this project.

### 2.2 Data collection

We compiled data for species and subspecies of songbirds across the world, from supplementary material in Barçante et al. (2017) and Wilman et al. (2014), and data mining from two online databases: IUCN Red List and BirdLife Data Zone (retrieved in November 2018). All entries were checked for nomenclature inconsistencies. Our universe consists of all 6,579 passerines in the IUCN Red List database, downloadable from their website https://www.iucnredlist.org/search after restricting (advanced) searches by taxonomy selecting, in the "search filters" option [Kingdom = Animalia; Phylum = Chordata; Class = Aves; Order = Passeriformes]. We associated four variables to each species: altitudinal migration status, primary habitat preference, foraging guild, and zoogeographic region.

A species was classified in our dataset as altitudinal migrant if its (common or scientific) name is listed in Barçante et al. (2017) either as altitudinal (238 species) or probable altitudinal migrant (592 species). BirdLife Data Zone provides, among many other information, the list of preferred breeding and nonbreeding habitats of a given species on the webpage http://datazone.birdlife.org/species/factsheet/common_name-scientific_name/details (where spaces are replaced by the character "‐" on its common and scientific names). Considering the great variety of habitats, we only used the major natural breeding habitat for each species and collapsed habitats into four major categories: dense habitat (forest + shrubland, 4,635 species), open habitat (grassland + savanna + open woodland + rocky areas, 563 species), water habitat (wetland + marine, 164 species), and generalist (species that occupied two or more major categories, 1,217 species). A total of 1,217 species occupied two or more major categories and were classified as generalists.

Foraging guild data were fetched from Willman et al. (2014), with species distributed among five categories: 754 frugivores/nectarivores, 547 seed/plant materials, 4,018 invertivores, 1,213 omnivores, and 20 vertebrates/fish/scavengers. Seventy‐one species had no information on Willman et al. (2014) and were classified with information from the Handbook of the Birds of the World Alive (del Hoyo, Elliott, Sargatal, Christie, & Kirwan, 2019) (47 species) or closest related species (24 species).

To build the zoogeographic region (Newton & Dale, 2001), we downloaded from IUCN Red List website 13 lists of Passeriformes, each with all Passeriformes observed on a specific "Land Region" (selected in the "search filters" option) and translated those regions to a reduced set of zoogeographical regions as follows: "Caribbean islands" = Neotropical, "Antarctica" = Neotropical, "East Asia" = Indomalayan, "Europe" = Palearctic, "Mesoamerica" = Neotropical, "North Africa" = Checked individually; "North America" = Neartic. "North Asia" = Palearctic. "Oceania" = Australian. "South America" = Neotropical, "South and Southeast Asia" = Indomalayan, "Sub‐Saharan Africa" = Afrotropical, "West and Central Asia" = Checked individually. Species residing on more than one zoogeographical region were classified as "Widespread" after manual investigation of their breeding distribution maps in the IUCN website. Our dataset consists of 1,298 Afrotropical (11% migrant), 816 Australasian (6% migrant), 1,422 Indomalayan (17% migrants), 288 Nearctic (31% migrant), 2,387 Neotropical (10% migrant), 342 Palearctic (20% migrant), and 26 Widespread (42% migrant) species.

### 2.3 Phylogeny

We downloaded the first 1,000 trees from Hackett backbone phylogenetic trees (Hackett et al., 2008). Hackett backbone phylogenetic trees are available from https://BirdTree.org (Jetz, Thomas, Joy, Hartmann, & Mooers, 2012). The trees were read in Rstudio (RStudio Team, 2016) using the *ape* package (Paradis & Schliep, 2018). We trimmed 4,105 species to only keep Passeriformes species using the drop.tip function in the *phytools* package (Revell, 2012). Using TreeAnnotator (Rambaut & Drummond, ), a maximum clade credibility tree was created with 1% burn‐in and mean heights. The final tree used in the analysis consisted of 5,888 species and 691 subspecies. Most subspecies are considered full species by IUCN (2019), but are not included in https://Birdtree.org phylogenies (Jetz et al., 2012). Since they were absent from the Hackett backbone phylogeny, subspecies were added to the tree by matching the genus and species names of the sister species (e.g., *Acrocephalus luscinius hiwae* matched with *Acrocephalus luscinius*) which created polytomies inside the phylogeny.

### 2.4 Statistical analysis

To examine evolutionary associations between altitudinal migration and life history characteristics, we used phylogenetic generalized least squares (pgls) analyses from the packages *ape* (Paradis & Schliep, 2018) and *nlme* (Pinheiro, Bates, DebRoy, & Sarkar, 2019). Brownian correlation and the maximum likelihood method were applied to each model. The models consisted of the response variable (altitudinal migration) coupled with each predictor individually (diet, habitat, and region), predictors paired together, or all predictors together. Two models also included an interaction; one between diet and region and one between habitat and region. The interaction was included to test whether the patterns of guild vary from one zoogeographical region to another as shown by Barçante et al. (2017); the same was applied to habitat. For the models with the interaction, we had to merge frugivore/nectarivore with seed/plant material and vertebrate/fish/scavenger with invertivore, resulting in three diet categories: herbivore, omnivore, and invertivore. We ranked the models using Akaike's information criterion (AIC). We considered the top models competitive if they differed by <4 AIC units.

## 3 RESULTS

**Figure 2:**
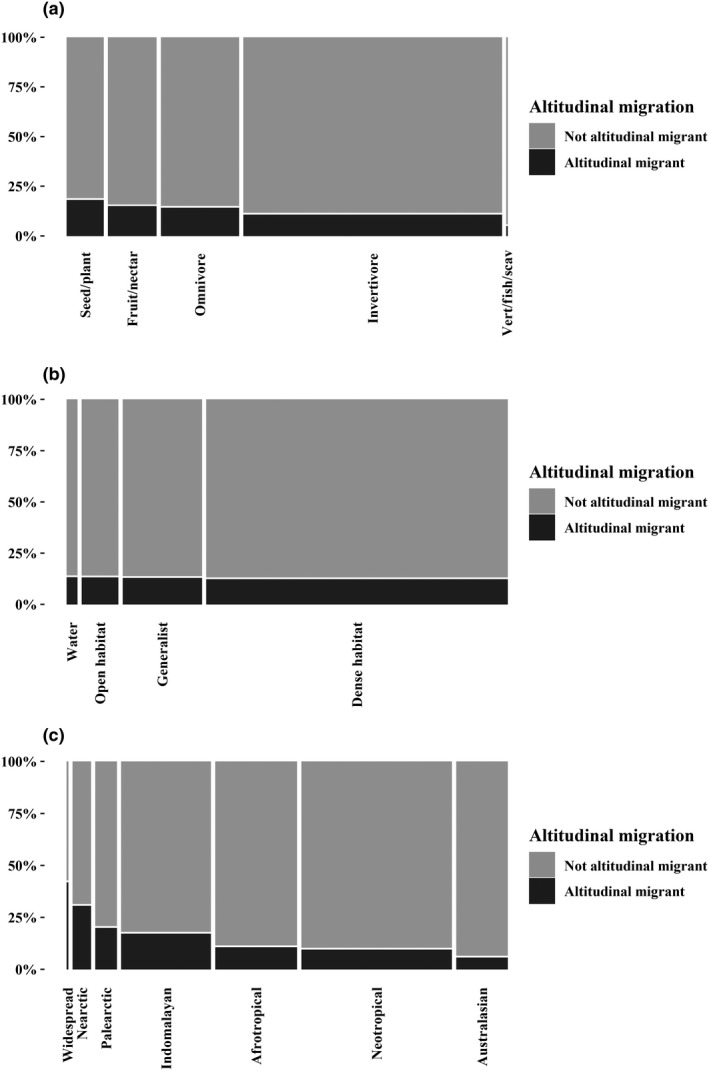
Mosaic plots representing the proportion of passerine species that are altitudinal migrant (black) or not (gray) for each foraging guild (a), habitat (b), and zoogeographical region when considering only breeding distribution (c). The width of the bars of the *x*‐axis indicates the proportion of species in each category

The best phylogenetic generalized least square model that predicted altitudinal migration included diet, region, an interaction between diet and region (Table 1 and Figure 2). The addition of habitat as a predictor did not improve the model's AIC. However, habitat was still associated with altitudinal migration (F_3_ = 3.98, *p* = .0076; Figure 2b), with more altitudinal migrants in open habitat than dense habitat, water, and generalist. When we examined the terms in the top‐ranked model, we found strong effects of foraging guild (F_2_ = 6.48, *p* = .0016; Figure 2a), region (F_6_ = 23.77, *p* < .0001; Figure 2c), and a foraging guild:region interaction (F_12_ = 10.05, *p* < .0001). The interaction model revealed that herbivore/widespread (*t* = 4.75, *p* < .0001), omnivore/Palearctic (*t* = 3.26, *p* = .0011), and omnivore and herbivore/Nearctic (*t* = 7.43, *p* < .0001, *t* = 4.43, *p* < .0001) species were more likely to exhibit altitudinal migration (Table 2).

**Table 1:** AIC results for each pgls model. The models are ranked from best to worst

Rank	Model	DF	AIC	ΔAIC
1	Diet + Region + Diet:Region	22	7,880.136	0
2	Diet + Habitat + Region	15	7,961.286	81.15
3	Diet + Region	12	7,975.992	95.856
4	Habitat + Region	11	7,980.389	100.253
5	Region	8	7,984.467	104.331
6	Diet + Habitat + Diet:Habitat	13	8,086.023	205.887
7	Diet	6	8,093.052	212.916
8	Diet + Habitat	9	8,097.665	217.529
9	Habitat	5	8,105.545	225.409

**Table 2:** *T*‐values for each variable included in the top‐ranked model Diet + Region + Diet:Region

	Value	Standard error	*t*‐Value	*p*‐Value
Intercept	0.077	0.53	0.14	.88
Omnivore	−0.0082	0.026	−0.32	.75
Herbivore	0.033	0.031	1.05	.29
Australasian	0.0091	0.033	0.27	.78
Indomalayan	0.021	0.026	0.80	.42
Nearctic	−0.069	0.054	−1.28	.20
Neotropical	−0.088	0.052	−1.70	.090
Palearctic	−0.051	0.035	−1.45	.15
Widespread	0.32	0.092	3.43	**.0006**
Omnivore: Australasian	0.032	0.043	0.74	.46
Herbivore: Australasian	−0.062	0.051	−1.21	.22
Omnivore: Indomalayan	−0.040	0.035	−1.16	.24
Herbivore: Indomalayan	−0.013	0.040	−0.32	.75
Omnivore: Nearctic	0.40	0.054	7.43	**<.0001**
Herbivore: Nearctic	0.27	0.061	4.43	**<.0001**
Omnivore: Neotropical	−0.017	0.037	−0.47	.64
Herbivore: Neotropical	0.054	0.045	1.19	.23
Omnivore: Palearctic	0.19	0.058	3.26	**.001**
Herbivore: Palearctic	0.073	0.061	1.20	.23
Omnivore: Widespread	0.13	0.26	0.50	.62
Herbivore: Widespread	0.59	0.12	4.75	**<.0001**

## 4 DISCUSSION

We explored two potential drivers of the evolution of altitudinal migration in passerines by conducting large‐scale phylogenetic comparative analyses. Our results indicate that foraging guild is evolutionarily associated with altitudinal migration, but this relationship varies across zoogeographic regions. Habitat did not appear to be strongly linked to the evolution of altitudinal migration.

Globally, species eating fruit/nectar or seed/plant material were more likely to exhibit altitudinal migration than omnivores and invertivores, despite the fact that most (61%) passerine birds are insectivorous. This observation follows most of the literature, which emphasizes that frugivorous altitudinal migrants should track fruit and flower abundance seasonally, particularly in Costa Rica (Blake & Loiselle, 2000; Boyle et al., 2011) and Nepal (Katuwal et al., 2016). Note that Barçante et al. (2017) in a study including all bird orders (not only Passeriformes) showed that invertivorous altitudinal migrants were more abundant around the world. Indeed, the number of invertivore species that are altitudinal migrants is higher than any other foraging guild; however, most Passeriformes eat invertebrates as their main diet and that foraging guild is by far the most speciose (4,018 of 6,579 species). However, the proportion of invertivorous altitudinal migrants was relatively low and we found no evolutionary association between altitudinal migration and invertivory for passerines, neither globally nor within regions. Note, however, that the classification of each species to one foraging guild is tricky because diet can vary through the seasons. Some birds might rely heavily on the intake of insects during the breeding season, but switch to fruits during the nonbreeding season. If food abundance is driving altitudinal migration, such a species may respond to insect abundance during the breeding season and fruit during the nonbreeding season. This situation likely reduced the effect of the patterns that we observed as we only considered the primary foraging guild (e.g., the main guild for each species depending on the distribution of the percentage among the diet categories, according to Wilman et al., 2014).

The regions that revealed an evolutionary association between altitudinal migration and foraging guild were the Nearctic, Palearctic, and Widespread. However, the foraging guilds associated with altitudinal migration differed between these regions. In the Nearctic, herbivore and omnivore species were more likely to be altitudinal migrants, a finding consistent with Boyle (2017). However, it is interesting that omnivorous species appear to be linked with altitudinal migration. This might support Chaves‐Campos (2004) and Levey (1988), who suggest that birds should follow fruit abundance during the nonbreeding season and insect abundance during the breeding season. Altitudinal migration would then be beneficial for omnivorous species. Omnivorous species are also linked to altitudinal migration in the Palearctic. Barçante et al. (2017) indicated that the proportion of frugivore/nectarivore species that are altitudinal migrant in the Palearctic was lower than expected; our results demonstrating a disproportionate number of omnivorous species agrees with their findings. For the species with a widespread distribution, herbivorous species were associated with altitudinal migration. This finding agrees with previous studies where herbivorous species have been indicated as altitudinal migrants all around the world (Blake & Loiselle, 2000; Guillaumet, Kuntz, Samuel, & Paxton, 2017; Katuwal et al., 2016; Kimura et al., 2001; but see Barçante et al., 2017). However, only 26 species are considered to have a widespread breeding distribution, so this interpretation should be taken with caution. For the other regions (Neotropical, Indomalayan, Afrotropical, and Australasian), foraging guild was not directly associated with altitudinal migration, potentially due to the vast complexity of tropical ecosystems.

Habitat was not associated with altitudinal migration in the top model, providing no additional information beyond what diet and region already provided. The proportion of altitudinal migrants present in each habitat were extremely similar (12%–13%); and no habitat had a disproportionate number of altitudinal migrants. However, habitat was still significant in the model with habitat only, with open habitats evolutionary associated with altitudinal migration. Thus, habitat may have played a role in the evolution of altitudinal migration, but foraging guild remains the main factor driving in our analysis.

Altitudinal migration is challenging to study in part because of the variability in the expression of the behavior. For instance, some populations within the same species are altitudinal migrants while the others are resident (Boyle, 2017; Green, Whitehorne, Middleton, & Morrissey, 2015). There is also variation in the propensity to engage in altitudinal migration among individuals within a population (Boyle, 2008b, 2017; Pratt et al., 2017; Rohwer et al., 2008) and within individuals across time (Hahn et al., 2004). In addition, most studies focus on the importance of altitudinal migration to birds moving to reach breeding grounds, but birds may also move up or downslope to reach molting grounds (Rohwer et al., 2008; Wiegardt et al., 2017). As such, this variation makes it extremely difficult to generalize and categorize birds as altitudinal migrants. We suggest that more studies are needed about specifics of altitudinal migration encompassing species not yet studied and these should begin to formalize distinctions between different types of altitudinal migration (e.g., facultative, breeding, and molting) to better understand this behavior and the drivers behind it (sensu Tonra & Reudink, 2018, formalization of molt‐migration). Molting and breeding are both energetically demanding and could both lead to strong selection for altitudinal movements. However, there are still some major differences between molting and breeding and those differences could be crucial in explaining the evolution of altitudinal migration.

Another limitation in our study is the lack of information for some regions (Barçante et al., 2017). We have confidence in the Nearctic since it has been well sampled and documented; approximately 31% species are altitudinal migrant which is the highest proportion within passerines (exception for Widespread). Otherwise, most studies in the Neotropics are concentrated in Costa Rica and there is limited research on altitudinal migration in the Afrotropical, Indomalayan, and Australasian regions (Barçante et al., 2017). Even the Palearctic, which is rich on research in avifauna, lacks data on altitudinal migration. This could mean either that altitudinal migration is rare in the Palearctic or that it has not been studied in depth.

The present study is the first to examine potential large‐scale drivers of the evolution of altitudinal migration in passerines. Altitudinal migration has evolved independently in different regions of the world under the different environmental pressures coupled with varying life history characteristics. Our results have reinforced the idea that diet (foraging guild) played a major role in the evolution of altitudinal migration. However, the relationship between diet and altitudinal migration is complex and varies across different regions in the world. Given the prevalence of this behavior across foraging guilds, diet is clearly not the only factor that drove the evolution of altitudinal migration, but rather the evolution of this trait was likely driven by an ensemble of factors.

## CONFLICT OF INTERESTS

None declared.

## AUTHORS CONTRIBUTION

M.W. Reudink, M.M. Vale, and C. Pageau conceived the project. C. Pageau wrote the manuscript, and all the authors edited it. C. Pageau, M. Shaikh, and M.W. Reudink wrote the code and conducted the statistical analysis. L. Barcante, M.A.S. Alves, M.A. de Menezes, and M.M. Vale aggregated the data.

### Open Research Badges

This article has earned an Open Data Badge for making publicly available the digitally‐shareable data necessary to reproduce the reported results. The data is available at https://doi.org/10.5061/dryad.jwstqjq5n.

## Data Availability

Data are accessible on Dryad (https://doi.org/10.5061/dryad.jwstqjq5n).

## References

[ece36126-bib-0001] Barçante, L. , Vale, M. M. , & Alves, M. A. S. (2017). Altitudinal migration by birds: A review of the literature and a comprehensive list of species. Journal of Field Ornithology, 88, 321–335. 10.1111/jofo.12234

[ece36126-bib-0002] Blake, J. G. , & Loiselle, B. A. (2000). Diversity of birds along an elevational gradient in the Cordillera Central, Costa Rica. The Auk, 117(3), 663–686. 10.1093/auk/117.3.663

[ece36126-bib-0003] Boyle, A. W. (2008a). Can variation in risk of nest predation explain altitudinal migration in tropical birds? Oecologia, 155, 397–403. 10.1007/s00442-007-0897-6 18188606

[ece36126-bib-0004] Boyle, A. W. (2008b). Partial migration in birds: Tests of three hypotheses in a tropical lekking frugivore. Journal of Animal Ecology, 77, 1122–1128. 10.1111/j.1365-2656.2008.01451.x 18657208

[ece36126-bib-0005] Boyle, A. W. (2010). Does food abundance explain altitudinal migration in a tropical frugivorous bird? Canadian Journal of Zoology, 88, 204–213. 10.1139/Z09-133

[ece36126-bib-0006] Boyle, A. W. (2017). Altitudinal bird migration in North America. The Auk, 134, 443–465. 10.1642/AUK-16-228.1

[ece36126-bib-0007] Boyle, A. W. , Conway, C. J. , & Bronstein, J. L. (2011). Why do some, but not all, tropical birds migrate? A comparative study of diet breadth and fruit preference. Evolutionary Ecology, 25, 219–236. 10.1007/s10682-010-9403-4

[ece36126-bib-0008] Boyle, A. W. , Norris, D. R. , & Guglielmo, C. G. (2010). Storms drive altitudinal migration in a tropical bird. Proceedings: Biological Sciences, 277, 2511–2519. 10.1098/rspb.2010.034 20375047 PMC2894928

[ece36126-bib-0009] Chaves‐Campos, J. (2004). Elevational movements of large frugivorous birds and temporal variation in abundance of fruits along an elevational gradient. Ornitologia Neotropical, 15(4), 433–445.

[ece36126-bib-0010] del Hoyo, J. , Elliott, A. , Sargatal, J. , Christie, D. A. , & Kirwan, G. (Eds.) (2019). Handbook of the birds of the world alive. Barcelona, Spain: Lynx Edicions Retrieved from http://www.hbw.com/

[ece36126-bib-0011] Green, D. J. , Whitehorne, I. B. J. , Middleton, H. A. , & Morrissey, C. A. (2015). Do American dippers obtain a survival benefit from altitudinal migration? PLoS ONE, 10(4), e0125734 10.1371/journal.pone.0125734 25905712 PMC4408061

[ece36126-bib-0012] Guillaumet, A. , Kuntz, W. A. , Samuel, M. D. , & Paxton, E. H. (2017). Altitudinal migration and the future of an iconic Hawaiian honeycreeper in response to climate change and management. Ecological Monographs, 87(3), 410–428. 10.1002/ecm.1253

[ece36126-bib-0013] Hackett, S. J. , Kimball, R. T. , Reddy, S. , Bowie, R. C. K. , Brau, E. L. , Braun, M. J. , … Yuri, T. (2008). A phylogenomic study of birds reveals their evolutionary history. Science, 320, 1763–1768. 10.1126/science.1157704 18583609

[ece36126-bib-0014] Hahn, T. P. , Sockman, K. W. , Nreuner, C. W. , & Morton, M. L. (2004). Facultative altitudinal movements by Mountain White‐crowned Sparrows (Zonotrichia Leucophrys Oriantha) in the Sierra Nevada. The Auk, 121(4), 1269–1281.

[ece36126-bib-0015] Hart, P. J. , Woodworth, B. L. , Camp, R. J. , Turner, K. , McClure, K. , Goodall, K. , … Samuel, M. (2011). Temporal variation in bird and resource abundance across an elevational gradient in Hawaii. The Auk, 128(1), 113–126. 10.1525/auk.2011.10031

[ece36126-bib-0016] Hayes, F. E. (1995). Definitions for migrant birds: What is a neotropical migrant? The Auk, 112(2), 521–523. 10.2307/4088747

[ece36126-bib-0017] IUCN (2019). The IUCN red list of threatened species. Version 2019–2. R core Team Retrieved from https://www.iucnredlist.org

[ece36126-bib-0018] Jetz, W. , Thomas, G. H. , Joy, J. B. , Hartmann, K. , & Mooers, A. O. (2012). The global diversity of birds in space and time. Nature, 491, 444–448. 10.1038/nature11631 23123857

[ece36126-bib-0019] Katuwal, H. M. , Basnet, K. , Khanal, B. , Devkota, S. , Ral, S. K. , Gajurel, J. P. , … Nobis, M. P. (2016). Seasonal changes in birds species and feeding guilds along elevational gradients of the Central Himlayans, Nepal. PLoS ONE, 11(7), e0158362 10.1371/journal.pone.0158362 27367903 PMC4930183

[ece36126-bib-0020] Kimura, K. , Yumoto, T. , & Kikuzawa, K. (2001). Fruiting phenology of fleshy‐fruited plants and seasonal dynamics of frugivorous birds in four vegetation zones on Mt Kinabalu, Borneo. Journal of Tropical Ecology, 17(6), 833–858.

[ece36126-bib-0021] Levey, D. J. (1988). Spatial and temporal variation in Costa Rican fruit and fruit‐eating bird abundance. Ecological Monographs, 58(4), 251–269. 10.2307/1942539

[ece36126-bib-0022] Loiselle, B. A. , & Blake, J. G. (1991). Temporal variation in birds and fruits along an elevational gradient in Costa Rica. Ecology, 72(1), 180–193. 10.2307/1938913

[ece36126-bib-0023] Mackas, R. H. , Green, D. J. , Whitehorne, I. B. J. , Fairhurst, E. N. , Middleton, H. A. , & Morrissey, C. A. (2010). Altitudinal migration in American Dippers (*Cinclus* *mexicanus*): Do migrants produce higher quality offspring? Canadian Journal of Zoology, 88, 369–377. 10.1139/Z10-013

[ece36126-bib-0024] Newton, I. , & Dale, L. (2001). A comparative analysis of the avifaunas of different zoogeographical regions. Journal of Zoology, 254, 207–218. 10.1017/S0952836901000723P

[ece36126-bib-0025] Papeş, M. , Peterson, A. T. , & Powell, G. V. N. (2012). Vegetation dynamics and avian seasonal migration: Clues from remotely sensed vegetation indices and ecological niche modelling. Journal of Biogeography, 39, 652–664. 10.1111/j.1365-2699.2011.02632.x

[ece36126-bib-0026] Paradis, E. , & Schliep, K. (2018). ape 5.3: An environment for modern phylogenetics and evolutionary analyses in R. Bioinformatics, 35, 526–528. 10.1093/bioinformatics/bty633 30016406

[ece36126-bib-0027] Pinheiro, J. , Bates, D. , DebRoy, S. , Sarkar, D. , &R Core Team (2019). nlme 3.1‐140: Linear and nonlinear mixed effects models. Retrieved from https://CRAN.R-project.org/package=nlme

[ece36126-bib-0028] Pratt, A. C. , Smith, K. T. , & Beck, J. L. (2017). Environmental cues used by Greater Sage‐Grouse to initiate altitudinal migration. The Auk, 134, 628–643. 10.1642/AUK-16-192.1

[ece36126-bib-0029] Rambaut, A. , & Drummond, A. J. (2018). TreeAnnotator v1.10.1: MCMC output analysis. Retrieved from http://beast.community/

[ece36126-bib-0030] Revell, L. J. (2012). phytools 0.6.99: An R package for phylogenetic comparative biology (and other things). Methods in Ecology and Evolution, 3, 217–223. 10.1111/j.2041-210X.2011.00169.x

[ece36126-bib-0031] Rohwer, V. G. , Rohwer, S. , & Barry, J. H. (2008). Molt scheduling of Western Neotropical migrants and up‐slope movement of Cassin's Vireo. The Condor, 110(2), 365–370. 10.1525/cond.2008.8321

[ece36126-bib-0032] Rosselli, L. (1994). The annual cycle of the White‐ruffed Manakin *Corapipo* *leucorrhoa*, a tropical frugivorous altitudinal migrant, and its food plants. Bird Conservation International, 4, 143–160. 10.1017/S0959270900002732

[ece36126-bib-0033] RStudio Team (2016). RStudio: Integrated Development for R. Boston, MA: RStudio, Inc Retrieved from https://www.rstudio.com/

[ece36126-bib-0034] Solorzano, S. , Castillo, S. , Valverde, T. , & Avila, L. (2000). Quetzal abundance in relation to fruit availability in a cloud forest in Southeastern Mexico. Biotropica, 32(3), 523–532. 10.1111/j.1744-7429.2000.tb00498.x

[ece36126-bib-0035] Stiles, F. G. (1988). Altitudinal movements of birds on the Caribbean slope of Costa Rica: Implications for conservation. Memoirs of the California Academy of Sciences, 12, 243–338.

[ece36126-bib-0036] Stiles, F. G. , & Clarke, D. A. (1989). Conservation of tropical rain forest birds: A case study from Costa Rica. American Birds, 43(3), 420–428.

[ece36126-bib-0037] Stotz, D. F. , Fitzpatrick, J. W. , Parker, T. A. , & Moskovits, D. K. (1996). Neotropical birds: Ecology and conservation. Chicago, IL: The University of Chicago Press.

[ece36126-bib-0038] Tonra, C. , & Reudink, M. W. (2018). Expanding the traditional definition of molt‐migration. The Auk, 135, 1123–1132. 10.1642/AUK-17-187.1

[ece36126-bib-0039] Wiegardt, A. , Wolfe, J. , Ralph, C. J. , Stephens, J. L. , & Alexander, J. (2017). Postbreeding elevational movements of western songbirds in Northern California and Southern Oregon. Ecology and Evolution, 7, 7750–7764. 10.1002/ece3.3326 29043031 PMC5632634

[ece36126-bib-0040] Willman, H. , Belmaker, J. , Simpson, J. , de la Rosa, C. , Rivadeneira, M. M. , & Jetz, W. (2014). Eltontraits 1.0: Species‐level foraging attributes of the world's birds and mammals. Ecology, 95(7), 2027 10.1890/13-1917.1

